# Multisectoral interventions for urban health in Africa: a mixed-methods systematic review

**DOI:** 10.1080/16549716.2024.2325726

**Published:** 2024-04-05

**Authors:** Meelan Thondoo, Ebele R. I. Mogo, Lambed Tatah, Monica Muti, Kim R. van Daalen, Trish Muzenda, Rachel Boscott, Omar Uwais, George Farmer, Adelaide Yue, Sarah Dalzell, Gudani Mukoma, Divya Bhagtani, Sostina Matina, Philip M. Dambisya, Kufre Okop, Charles Ebikeme, Lisa Micklesfield, Tolu Oni

**Affiliations:** a MRC Epidemiology Unit, School of Clinical Medicine, University of Cambridge, Cambridge, UK; b Barcelona Institute for Global Health (ISGlobal), Centre for Research in Environmental Epidemiology (CREAL), Barcelona, Spain; c SA MRC/Wits Developmental Pathways for Health Research Unit, Department of Paediatrics, Faculty of Health Sciences, School of Clinical Medicine, University of the Witwatersrand, Johannesburg, South Africa; d British Heart Foundation Cardiovascular Epidemiology Unit, Department of Public Health and Primary Care, University of Cambridge, Cambridge, UK; e Victor Phillip Dahdaleh Heart and Lung Research Institute, University of Cambridge, Cambridge, UK; f Barcelona Supercomputing Center (BSC), Department of Earth Sciences, Barcelona, Spain; g Department of Biokinetics, Recreation and Sport Science, Faculty of Health Sciences, University of Venda, Thohoyandou, South Africa; h Health Policy and Systems Division, School of Public Health and Family Medicine, University of Cape Town, Cape Town, South Africa; i Centre for Innovation in Learning and Teaching, University of Cape Town, Cape Town, South Africa; j Chronic Disease Initiative for Africa (CDIA), Department of Medicine, University of Cape Town, Cape Town, South Africa; k LSE Health, Department of Health Policy, London School of Economics and Political Science, London, UK

**Keywords:** Urban health, multisectoral action, Africa, cities, non-communicable diseases

## Abstract

Increasing evidence suggests that urban health objectives are best achieved through a multisectoral approach. This approach requires multiple sectors to consider health and well-being as a central aspect of their policy development and implementation, recognising that numerous determinants of health lie outside (or beyond the confines of) the health sector. However, collaboration across sectors remains scarce and multisectoral interventions to support health are lacking in Africa. To address this gap in research, we conducted a mixed-method systematic review of multisectoral interventions aimed at enhancing health, with a particular focus on non-communicable diseases in urban African settings. Africa is the world’s fastest urbanising region, making it a critical context in which to examine the impact of multisectoral approaches to improve health. This systematic review provides a valuable overview of current knowledge on multisectoral urban health interventions and enables the identification of existing knowledge gaps, and consequently, avenues for future research. We searched four academic databases (PubMed, Scopus, Web of Science, Global Health) for evidence dated 1989–2019 and identified grey literature from expert input. We identified 53 articles (17 quantitative, 20 qualitative, 12 mixed methods) involving collaborations across 22 sectors and 16 African countries. The principle guiding the majority of the multisectoral interventions was community health equity (39.6%), followed by healthy cities and healthy urban governance principles (32.1%). Targeted health outcomes were diverse, spanning behaviour, environmental and active participation from communities. With only 2% of all studies focusing on health equity as an outcome and with 47% of studies published by first authors located outside Africa, this review underlines the need for future research to prioritise equity both in terms of research outcomes and processes. A synthesised framework of seven interconnected components showcases an ecosystem on multisectoral interventions for urban health that can be examined in the future research in African urban settings that can benefit the health of people and the planet.

**Paper Context**
- **Main findings:** Multisectoral interventions were identified in 27.8% of African countries in the African Union, targeted at major cities with five sectors present at all intervention stages: academia or research, agriculture, government, health, and non-governmental.
- **Added knowledge:** We propose a synthesised framework showcasing an ecosystem on multisectoral interventions for urban health that can guide future research in African urban settings.
- **Global health impact for policy and action:** This study reveals a crucial gap in evidence on evaluating the long-term impact of multisectoral interventions and calls for partnerships involving various sectors and robust community engagement to effectively deliver and sustain health-promoting policies and actions.

## Introduction

By 2050, it estimated that 68% of the global population will be living in cities, with most urban residents residing in low- and middle-income countries (LMICs) [1]; as such urban environments play an increasingly pivotal role in the health and wellbeing of people and the planet. Projections indicate that Africa’s urban population will triple from about 395 million people in 2010 to approximately 1.339 billion people by 2050, which equals one-fifth of the world’s projected urban population in 2050 [2]. African cities such as Kinshasa (Democratic Republic of Congo), Accra (Ghana), Lagos (Nigeria), Khartoum (Sudan), Johannesburg-Pretoria (South Africa), Nairobi (Kenya) and Cairo (Egypt) all have populations surpassing 10 million inhabitants, while Dar-es-salaam (Tanzania) and Luanda (Angola) will be joining these ranks soon [3].

As reflected by the recent COVID-19 pandemic, urbanisation can have negative and complex impacts on human health. Urban populations are exposed to unhealthy factors that contribute to a rise in chronic diseases, risk factors such as physical inactivity and unhealthy dietary behaviours, and unequal exposure to socio-economic inequities [4]. This urban penalty includes the double burden of non-communicable diseases (NCDs) and infectious diseases [5]. African cities are becoming more obesogenic, as the food environment increasingly offers low nutrient energy-dense (LNED) foods, and is characterised by the unbridled marketing of health-harming commodities and unhealthy foods, poor access to safe, inclusive physical activity and recreational infrastructure [6,7]. The indiscriminate importation of health-harming commodities such as tobacco, alcohol [8] and ultra-processed foods contributes to these exposures. When combined with unplanned urban development and rising poverty, these exposures create higher risks for obesity and other NCDs [9]. As a result, in Africa, NCD prevalence is projected to increase by 27% on the continent as urbanisation continues, with estimated NCD deaths expected to increase from 30.8 million in 2015 to 41.8 million by 2030 [10–12].

**Figure 1.**
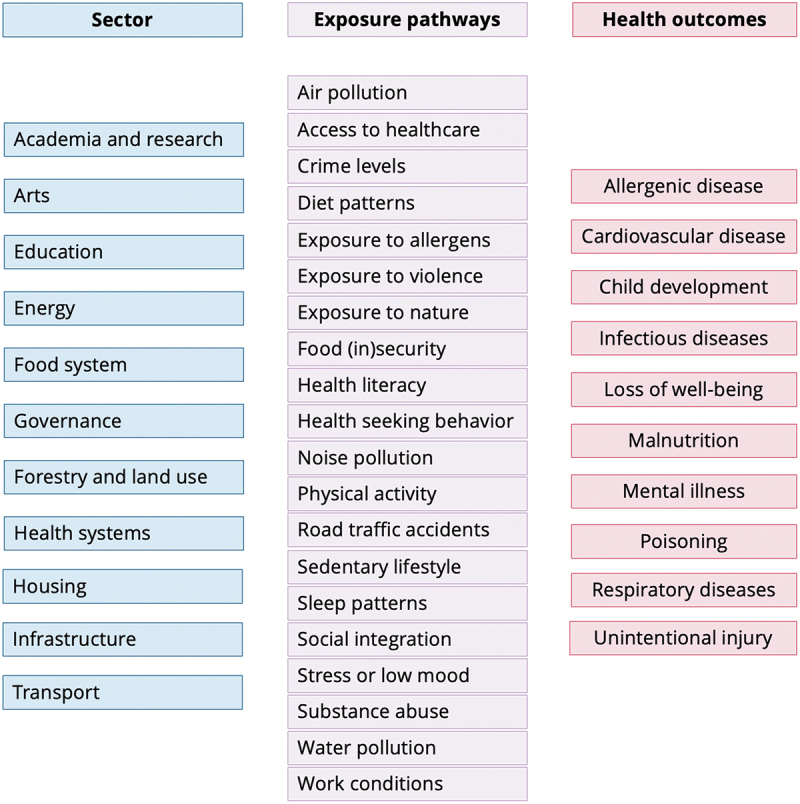
Examples from the included studies, of different urban sectors, exposure pathways and health outcomes affecting health in African cities.

Evidence shows that to tackle multifaceted health challenges, it is crucial to acknowledge that many determinants and drivers of health lie outside the health sector [13]. This is particularly true in urban settings as a wide number of factors across different sectors may interact and synergise to affect disease and mortality. Cities are particularly vulnerable to water scarcity, energy poverty, and food insecurity due to climate change, the growing frequency and intensity of extreme climate and weather events, and socio-political unrest. Interventions in one sector targeted towards specific health problems can affect other health outcomes, and often in different ways (see Figure 1 for examples from the included studies, of different urban sectors, exposure pathways and health outcomes affecting health in African cities). Thus, health-driven initiative interventions or activities that improve the social, built and communal aspects of urban environments across sectorsare crucial to improve urban residents’ health and wellbeing.

This systematic review aims to synthesise evidence on interventions utilising collaborations across different sectors (i.e. multisectoral interventions) in African cities to improve health by simultaneously addressing complex issues that cannot be addressed by a single sector alone. For this study, we use NCDs as a lens through which urban health can be addressed. It provides a snapshot of the types of multi-stakeholder collaborations that exist in urban health, and to allow for the identification of existing knowledge gaps and, consequently, avenues for future research that can inform policy and practice. First, this Systematic Literature Review (SLR) provides a thematically and methodologically organised, state-of-the-art classification of multisectoral interventions with respect to their application sectors, limitations, and recommendations. Second, based on the findings of the SLR, we propose a synthesising framework to detail potential themes that require scholarly attention to advance the current body of knowledge.

Findings from this systematic review are intended to inform city actors and decision makers on the wide range of existing population-level multi-sectoral interventions, and what makes them work or fail, for which target populations, and under which circumstances. The framework presented here, aims to recast current views on multisectoral urban health intervention research in LMICs and suggests new areas for investigation. This review specifically addresses the following research questions:

1. What is the scope of multisectoral interventions that exist and have been published in literature to improve health, and decrease NCD prevalence in African cities?
2. Which sectors are involved at the different stages of multisectoral interventions to improve urban health?
3. What are the guiding principles, targeted health outcomes and measurements of the impact of these outcomes in multisectoral interventions?
4. What factors influence the success or effectiveness of multisectoral interventions?
5. What are the different components of a framework that can guide future research into multisectoral interventions to improve urban health?

## Methods

This systematic review applied mixed-method approaches to integrate qualitative and quantitative findings of relevance to multi-sectoral interventions for NCD prevention in urban contexts. The focus, inclusion criteria and framework for this study were informed by a stakeholder engagement workshop organised in 2019 with multi-sector decision-makers from East, West, Central and South Africa [14].

### Search strategy

This mixed-methods systematic review was conducted following the PRISMA guidelines [15] (Supplementary file 0: PRISMA Checklist), and the protocol was prospectively registered with PROSPERO (CRD42020189285) [16]. The search was conducted between September and December 2019. The search strategy aimed at identifying qualitative, quantitative, and mixed-methods studies on multi-sectoral interventions to improve urban health in African cities. Four academic databases (PubMed, Scopus, Web of Science and Global Health) were searched from inception of the database through to 21 December 2019 using predetermined medical subject headings (MeSH) terms (Supplementary file 1: Search Strategy).

We included all studies that were 1) focusing on multi-sectoral interventions in cities (see section 2.2), 2) quantitative, qualitative or mixed-methods studies that contained primary or secondary data, 3) published since 1990 and 4) published in any language (i.e. no language restrictions). Note, 1990 was defined as the cut-off point for the search because it marked the beginning of the promotion of the concept of healthy cities [17]. We excluded 1) literature reviews and narrative overviews which described multi-sectoral initiatives and did not analyse primary or secondary empirical data, 2) summaries and articles for which the full text was not available, 3) commentaries and opinion pieces which did not have primary data, 4) conference proceedings and 5) interventions that focused on managing existing NCDs (e.g. interventions to manage disability due to stroke); clinical interventions addressing NCD prevention that did not involve any partnerships (e.g. hospital-based interventions) as well as interventions that may have included a component of NCD prevention but did not explicitly state this (e.g. broader water and sanitation (WASH) interventions) (see Supplementary file 2: Inclusion and Exclusion Criteria).

We uploaded all the studies identified with the search strategy into the Rayyan software, a digital systematic review platform to review, select, and conduct quality assessment of studies. Titles, abstracts, and full-text were double-screened according to the inclusion and exclusion criteria. Where conflicts arose in study selection, two or more investigators involved in the screening clarified and resolved them. All non-English records were reviewed by a native or fluent speaker of the research team, which included members fluent in several languages such as English, French, German, Spanish and Portuguese. We also performed forward and backward screening of the included studies using Google Scholar. Grey literature was recommended from key contacts in government and non-governmental agencies and academic topic experts during a consultation exercise in 2019 [14] and from the Global Diet and Activity Research collaborators across the partner universities.

### Theoretical framework and definitions

The World Health Organization (WHO) and UN-Habitat framework for integrating health into urban and territorial planning [18] informed our study. This framework describes four strategies to integrate health into decision-making using four entry points – by setting, by sector, by principles and by outcomes. Following the United Nations Statistical Commission’s (UNSC) international definition, cities were defined as settlements with a population of at least 50,000 dwellers [19] who live in contiguous dense grid cells with more than 1,500 inhabitants per square kilometre [20] African cities were cities from a list of African Union member states (see Supplementary file 3: Included Countries).

### Multisectoral interventions

While intersectoral work also involves collaboration and coordination between different sectors, it focuses on a particular issue within a defined context (an economy or society) and does not require the involvement of a broader range of stakeholders (non-governmental organisations, community groups and experts), which is often the case for multi-sectoral work [21]. For instance, if a particular issue within the healthcare system (consisting of hospitals, primary care, and public health agencies) was to be solved, the result of intersectoral work could be the provision of coordinated care to patients with chronic diseases through the collaboration of a hospital and a primary care clinic. Multi-sectoral work, in contrast, would require the collaborations of multiple sectors within the healthcare system as well as other sectors outside of healthcare, such as education, transportation, and housing. Such an intervention would likely involve the participation of non-governmental organisations, community groups, and other stakeholders, leading to the involvement of the education sector (for example) to address care of patients with chronic diseases by providing healthy food options in the workplace and promoting physical activity.

We included multi-sectoral interventions focused on NCD prevention in urban African contexts. These included interventions are characterised by the involvement of multiple sectors (i.e. specific areas of responsibility or activity within a government or a community) to achieve one of the following aims: i) improvements to the built and natural environment in urban (including informal) settings, ii) building partnerships across sectors to address the health and wellbeing of the urban population, iii) improvements to the social infrastructure, participation and empowerment of community members, and/or iv) improving equity in involvement, access and impact to existing urban health initiatives. Studies targeting improvement in commercial environments were not included due to time and scope limitations of the review. Studies describing multi-sectoral interventions were classified into five categories: planning an intervention, forming a collaboration, implementing an intervention, measuring impact of an intervention, and monitoring or evaluation of an intervention.

### Targeted health outcomes

The target health outcomes of the multi-sectoral interventions included within this study (i.e. those that met the above inclusion criteria) were classified into five categories (see Supplementary file 4: Data extraction template):

- Health behaviours
- Improved access to health-promoting services
- Providing social infrastructure to improve participation and empowerment of community members
- Health profiles and disease outcomes
- Health equity

### Study population

The target population of all multi-sectoral interventions were residents of cities in Africa. There was no limit to the age, gender, ethnicity, or other social identifiers of the populations targeted within this review.

### Data extraction

A template was used to extract data from all included studies (see Supplementary file 4: Data extraction template). This was designed, piloted and validated by two researchers. Validation was achieved by each extracting 10% of the included articles, comparing results and adapting the template accordingly. Subsequently, other researchers (see Supplementary file 5: Researcher roles) double-extracted data using the validated template. Emerging conflicts were resolved among authors by consensus. Extracted information included author, publication year, study title, study design, study and/or target population, type of intervention, location or setting of intervention, underlying principle of the intervention (i.e. the fundamental values and concept that shape the way that urban health is approached and addressed, including health equity, sustainability, and intersectionality), entry point (the specific aspect or factor leveraged as a starting point for promoting healthy, including setting, sector, and outcomes) driving the intervention, targeted outcome of the intervention, factors acting as barriers or facilitators of the intervention and lessons learnt. One of the outcomes reviewed included ‘partnerships’ (see Findings section on partnerships and integration) that authors considered ‘present or not present’ in a general sense, rather than a methodological category through which to report the studies. The authors considered partnerships as a framework for enabling actions operating at different individual to interorganisational levels, and putting in place organisational preconditions, a functional well-structured team and/or actively building interpersonal and individual collaborative capacity [22].

### Quality appraisal methods

The quantitative (i.e. for trial, cohort and cross-sectional studies) and qualitative checklists of the Critical Appraisal Skills Programme (CASP) were used for quality assessment of the included studies [23] (see Supplementary file 6: CASP Qualitative checklist). Mixed-methods studies were assessed with both the relevant quantitative and qualitative CASP checklists. The CASP checklist was modified to accommodate cross-sectional studies. Whilst the checklists could be converted into a summary score, this approach can oversimplify important differences in bias, confounding and overall quality of individual studies. Thus, no overall score was assigned. No thresholds for good, fair, or poor quality were used, nor were studies excluded based on their quality assessment. Instead, more robust studies were prioritised in the interpretation stage and the information synthesis. The CASP appraisal tool was predominantly used to support the identification of recurring limitations in studies exploring multi-sectoral interventions. This approach was used mirroring a recent study conducted by research team members investigating the socio-economic dimensions of public space use for transport and its implications for health and wellbeing in African cities [24].

### Data analysis and synthesis

Data analysis and synthesis were informed by iterative discussions among the authors. Given the heterogeneity in study designs, analytic units, and assessment methods used among the included studies, no meta-analyses were performed. Thus, quantitative results were discussed descriptively, and further included in a thematic synthesis [25]. A parallel convergent design was applied to a thematic synthesis approach to compare qualitative and quantitative findings concurrently and allowed findings to simultaneously enrich one another [26]. A thematic synthesis approach is appropriate for synthesising evidence to inform interventions considering [27,28], it allows for the integration of mixed-methods data into various categories and transformation of data into emergent themes, and it can be theory-driven, or in our case, data-driven [29]. The four steps of thematic synthesis are summarised below (see Table 1).

**Table 1.** The thematic synthesis of the included studies.

Step	Data	Description	Key questions to explore	Output
1. Data coding	Key extraction domains as per the extraction tool CASP tools	Coding of qualitative data Descriptive summary tables of quantitative data	What components, partners, challenges, opportunities, and outcomes are associated with multi-sectoral initiatives to address NCD risks in African cities? What quality issues need to be addressed in future transdisciplinary research?	Qualitative codes Descriptive quantitative data
2. Data translation to generate overarching themes	Quantitative data Qualitative data	Translation of the combined quantitative and qualitative data codes into themes	What components, partners, challenges, opportunities, and outcomes are associated with multi-sectoral initiatives to address NCD risks in African cities? What quality issues need to be addressed in future transdisciplinary research?	Overarching themes from the combined qualitative and quantitative data
3. Data synthesis to provide recommendations	Quantitative data Qualitative data Grey literature	Transformation of the overarching themes into priorities into policy & intervention recommendations	What are the implications of the research findings for future multi-sectoral interventions to address NCDs in Africa?	Provisional recommendations for policy, action, and future transdisciplinary research
4. Validation of the emergent priorities	Quantitative data Qualitative data Grey literature	Stakeholder workshop with research steering group members and multisectoral policy and grassroots actors in urban African cities involving: (i) Presentation of analytic approach (ii) Presentation of the overarching themes identified (iii) Presentation of preliminary recommendations (iv) Invitation for comments which may agree, disagree, expound on, or add emergent considerations that need to be captured	What are the implications of the research findings for future multi-sectoral interventions to address NCDs in Africa?	Finalised recommendations for policy, action, and future transdisciplinary research

## Findings

**Figure 2.**
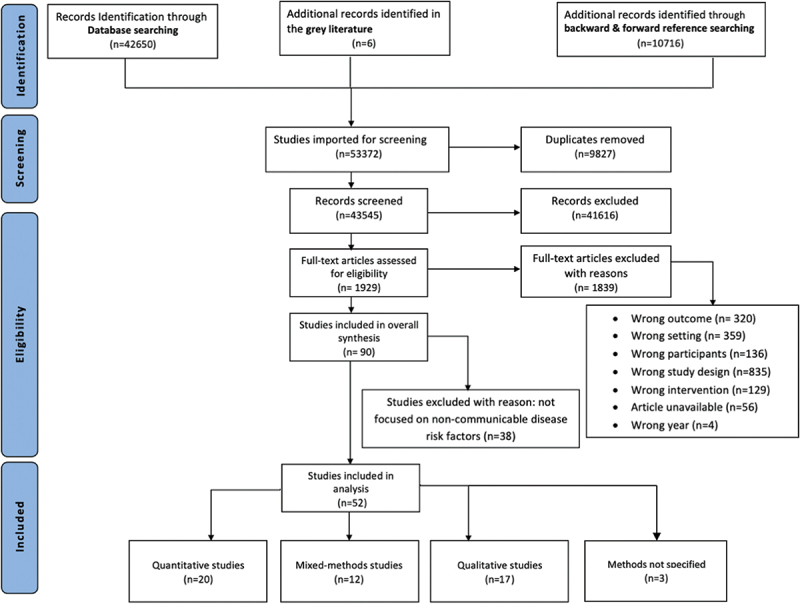
PRISMA flow diagram illustrating the study selection.

The search strategy yielded 53,372 records. After excluding duplicates, 43,545 records were screened by title and abstract, and 1,929 in full text. In total, 52 records were included to synthesise and pool results in this mixed-methods systematic literature review (Figure 2).

### Study characteristics

Of the 52 eligible articles (see Table 7), 20 (38.4%) were qualitative studies, 17 (32.7%) were quantitative, and 12 (22.6%) were mixed methods studies (see Table 7 for the list of 52 studies). Most studies, 64% (*n* = 34), were published between 2015 and 2019 (see Table 2). Overall, 30% of the 54 African Union countries were represented in the studies reviewed (Table 2). Four studies were on multi-country sites. Most studies focused on South Africa (*n* = 21) followed by Kenya (*n* = 6). In both countries, a larger number of interventions took place in major cities, such as Cape Town [32,39,46,50,60,64–66,70] and Nairobi [35,51,52,54,76].

**Table 2.** Overview of studies.

Methodological design	N. of articles	Setting	N. of articles
Qualitative	20	Formal	18
Mixed methods	12	Informal	8
Quantitative	17	Formal and Informal	2
Not specified	3	Not specified	24
Country of Implementation	N. of articles	Year of publication	N. of articles
Burkina Faso	1	2002	1
Egypt	3	2005	1
Ethiopia	3	2006	2
Ghana	3	2007	2
Ghana, Nigeria, Sierra Leone	1	2008	1
Kenya	6	2009	1
Kenya, Chile	1	2010	3
Madagascar, Burkina Faso	1	2011	1
Namibia	1	2012	1
Nigeria	2	2013	3
Senegal	3	2014	2
South Africa	21	2015	7
Eswatini	1	2016	7
Tanzania	2	2017	7
Uganda, Kenya	1	2018	9
UK, South Africa	1	2019	4
Zimbabwe	1		

### Data sources and author affiliations

There is a disparity in the geographic locations of the affiliations of the first and last authors (Table 3) with 47% (*n* = 25) of studies with first authors located outside of the continent. The main data sources for the articles were academic institutions (*n* = 13) followed by government data sources (*n* = 3), NGO (*n* = 1), other (*n* = 1), private (*n* = 1) and research institutes (*n* = 1).

**Table 3.** Study location and location of affiliations of the first and last authors.

Study location	First author location	Last author location
Burkina Faso (2) [38,40]	France (2) [38,40]	France (1) [40] Canada (1) [38]
Egypt (3) [31,45,47]	Egypt (3) [31,45,47]	Egypt (3) [31,45,47]
Eswatini (1) [58]	Switzerland (1) [58]	Eswatini (1) [58]
Ethiopia (3) [55,73,77]	Canada (1) [77] Ethiopia (2) [55,73]	Ethiopia (3) [55,73,77]
Ghana (4) [30,34,37,44]	Ghana (2) [34,44] Switzerland (1) [30] United States (1) [37]	Ghana (2) [34,44] the Netherlands (1) [44] United Kingdom (1) [30] United States (1) [37]
Kenya (8) [35,36,51,52,54,72,74,78]	Canada (1) [54] Kenya (2) [36,74] United Kingdom (2) [35,78] United States (3) [51,52,72]	Canada (1) [54] Chile (1) [78] the Netherlands (1) [74] Kenya (1) [35] United Kingdom (2) [35,36] United States (3) [51,52,72]
Madagascar (1) [40]	France (1) [40]	France (1) [40]
Namibia (1) [49]	Namibia (1) [49]	Namibia (1) [49]
Nigeria (3) [44,59,69]	Ghana (1) [44] Nigeria (1) [59] United States (1) [69]	Ghana (1) [44] the Netherlands (1) [44] Nigeria (1) [59] United States (1) [69]
Senegal (3) (Gartner et al., 2006) [79,80][NO_PRINTED_FORM]	France (3) [53,79,80]	France (3) [53,79,80]
Sierra Leone (1) [44]	Ghana (1) [44]	Ghana (1) [44] the Netherlands (1) [44]
South Africa (22) [22,32,33,39,41–43,46,48,50,57,60,62,64–68,70,71,75,81]	Nigeria (1) [33] United States (6) [32,46,50,60,68,75] South Africa (17) [22,32,39,41–43,48,57,60,62,64–67,70,71,81]	Australia (1) [66] Belgium (1) [62] Germany (1) [32] Nigeria (1) [33] South Africa (13) [22,32,39,41,42,48,57,60,64,67,69,71,73] United Kingdom (2) [43,74] United States (5) [32,46,50,66,78]
Tanzania (2) [56,61]	Tanzania (1) [61] United Kingdom (1) [56]	Tanzania (1) [61] United Kingdom (2) [56,61]
Uganda (1) [54]	Canada (1) [54]	Canada (1) [54]
United Kingdom (1) [71]	South Africa (1) [71]	United Kingdom (1) [71]
Zimbabwe (1) [63]	Zimbabwe (1) [63]	Zimbabwe (1) [63]

### Sectors involved

In total 22 sectors were identified across the multisectoral interventions across the included studies. The sectors were listed and reviewed based on the stages of the intervention where they intervened as per Table 4. Five sectors were present at all intervention stages: academia or research, agriculture, government, health, and non-governmental. Six sectors were present at three stages: community, environment, infrastructure, media, social services, and workplace. Five sectors were present at one stage only: arts, energy, individual, religion, and urban design (Table 4).

**Table 4.** Sectors involved and stages of multisectoral intervention development.

	Design	Funding	Implementation	Monitoring & Evaluation	Advocacy
Academia/Research	x	x	x	x	x
Agriculture	x	x	x	x	x
Arts	x				
Community	x		x	x	
Drug retail	x			x	
Education	x		x		
Energy	x			x	
Environment	x	x		x	
Finance	x	x			
Government	x	x	x	x	x
Health	x	x	x	x	x
Individual		x			
Infrastructure	x		x	x	
Insurance			x	x	
Law	x		x		
Media	x		x		x
Non-governmental	x	x	x	x	x
Religion	x				x
Social services	x	x		x	
Transport	x			x	
Urban design	x				
Workplace		x		x	x

### Guiding principles, targeted health outcomes and measurement of impact

Across 52 studies, we identified nine guiding principles (Table 5), six health outcomes of interest (Table 6), and no measurement of the impact of the interventions on these outcomes.

**Table 5.** Principles guiding the implementation of interventions.

Community health equity (39.6%)	[30,34,35,37,40,47,52–54,56–58,62,64,69,70,73,74,78–80]
Healthy cities and healthy urban governance (32.1%)	[35,36,38–40,43,45–48,51,55,64–66,71,74,78]
Community participation (19%)	[22,35,38–40,43,46,51,65,78]
Environmental sustainability (11.3%)	[22,33,41,44,45,52]
Food security and healthy eating (9.4%)	[63,68,72,80,82]
Social justice and economic well-being (7.5%)	[35,47,52,60,64,68,78]
Health promotion and prevention (5.7%)	[32,42,50,59–61,78,80]
Road danger reduction (3.7%)	[38,67]
Age friendly cities (1.9%)	[38]

**Table 6.** Targeted health outcomes.

Healthy Behaviour (23%)	[30–32,35,36,39,40,42,48,50,53,57,65,68–70,73,79,83]
Health profiles and disease outcomes (30%)	[30,32,33,37,47,51,56,57,59–61,63,70,73,77,78]
Improving the environment (physical and natural) (23%)	[38,41,44,45,47,52,57,64,66,67,72,81]
Providing social infrastructure to improve participation and empowerment of community members (13%)	[22,46,51,54,56,74,75]
Improving access to health-promoting services (11%)	[49,55,62,63,69,71]
Health equity (4%)	[34,58]

Targeted outcomes were diverse, spanning behaviour, environmental and active participation from communities (Table 6).

### Factors influencing success

This section addresses four different factors (a-d) that were reported to influence the success of multisectoral interventions in this review.

#### Administrative processes

Administrative processes were identified as a crucial factor in the initial and long-term success of multisectoral interventions. Processes such as the deployment and marketing of interventions as well as access to basic services (water and electricity) and infrastructure are needed for the effective implementation of interventions [37,40,44,48]. For example, delays in the delivery of services or resources in Cape Town (South Africa) and Accra (Ghana) [37,39] and poor means for follow-up on particular interventions [66] can contribute to whether tasks that are necessary to run the interventions are completed or not. Good administration can also enable effective follow-up with project participants, as shown in Thekwini (South Africa) and Dar es Salaam (Tanzania) [61,81] and the lack of it can complicate follow-up and assessment of intervention’s impact. In one case, despite the initial feasibility of a multisectoral initiative enabling private sector retail stores to screen community members for hypertension, only 46% of people who were screened were reachable on their provided numbers during follow-up [61]. Administrative hurdles can also drive attrition through inadequate consideration of existing time pressures on project implementers [30,48,64]. Where available, inclusive, and diverse administrative leadership was an asset to project implementation in Cape Town [66] and stood in stark contrast with interventions facing poor availability of administrative leadership to drive projects as reflected in Nairobi [52] or those experiencing fraud [64].

#### Local capacity and resources

Local capacity and access to resources were important for the long-term success of interventions. Funding and critical resources such as infrastructure for transport, medical supplies, and mobility, as well as tools to measure and evaluate the impact of interventions, need to be considered beyond the initial feasibility testing for the effective implementation of multisectoral interventions [31,55,58]. We found examples where sustainability of interventions was hindered by centralisation of power within development organisations without adequate investment of resources and capacity into local governments or communities [44]. The ability to measure and adequately plan for the right number of resources needed can support the implementation of interventions, including formative research projects, evaluation, and community-based participatory action methods [39]. This is particularly true when considering the long-term impact of interventions in LMICs contexts where urban populations can be highly mobile due to resource and economic pressures [79]. Comprehensive and appropriate measurement and evaluation efforts can support the inclusion of populations that are most likely to be excluded from interventions due to lack of access to services and reduced mobility [55] or individuals that work away from the community or city of interest, making them harder to target for inclusion in multi-sectoral interventions [69].

#### Partnerships and integration

Partnerships are crucial for multi-sectoral interventions. A wide range of sectors that partnered collaboratively in the implementation of interventions were identified. This included, for example, partnerships such as 1) a partnership between government health facilities and private sector drug retail outlets to screen for hypertension and make referrals for further treatment [61], 2) a partnership between population, employment, housing and land use sectors to develop a sustainable transport initiative [45], 3) a partnership between policymakers and community groups to address malnutrition [78] and 4) a partnership between local police officers and researchers to collect data and support surveillance of road traffic injuries [38]. Yet, establishing partnerships alone is not sufficient to ensure the success of multisectoral interventions. There is a need to sustainably integrate those partnerships into each other [62] which requires the development of collaborative, interpersonal, and organisational structures and capacities. In some cases, the Ministry of Health and existing health centres were helpful partners to each other in implementing multisectoral interventions [74], while the adoption of interventions by the government sectors and committees helped with their scaling and continuation [82]. When interventions include substantial private sector involvement, particularly private medical providers such as retail outlets and insurers, it is important to explore how incentives can be best aligned to ensure that all (especially the resource poor) have access to the intervention and its benefits [61,69].

#### Community engagement

Communities and the groups that represent them, such as civil society groups and non-governmental organisations, are critical partners in multisectoral initiatives. However, while there are several instances that confirm the important role of community volunteers in intervention delivery, long-term interest of these volunteers can wane [41,43,77]. To sustain interest and engagement of community volunteers, it is crucial to understand communities’ preferences, needs, and capacity. In one instance, older community members were targeted as volunteers for an irrigation project aimed at improving food security, but they identified their role as being too labour intensive and consequently dropped out of the intervention [63]. In another case, communities did not feel that interventions addressed their pressing daily and economic needs and were therefore unwilling to be engaged [36]. Appropriately building on the knowledge, expertise, and experience held by local communities is crucial in ensuring that community engagement leads to successful interventions and their evaluation [73]. Community members can help with adapting interventions to a specific context or setting of the target population of interest. For example, church members may support interventions by designing health interventions within the context of the ethos and values of their church [43]. Community members can also play a crucial role in getting government buy-in into the intervention as early as possible and to ensure that sufficient time is available for different partnerships with community groups to be established and grow [70]. Community health volunteers and extension workers are often lauded for their positive impact on health initiatives, so it is crucial to consider the strain that interventions may apply on their already limited time and resources over time [36]. In one instance, the presence of community champions in a nutrition intervention and their absence from a similar intervention in a different context is suggested to have contributed to the failure of the latter project by limiting opportunities to secure funding and integrate the project into the community [68]. Finally, language should be underlined as a determining factor to success. Local communities are most likely to engage in their local tongue (e.g. isiXhosa instead of English) [65].

### Framework for future research on multi-sectoral interventions

**Figure 3.**
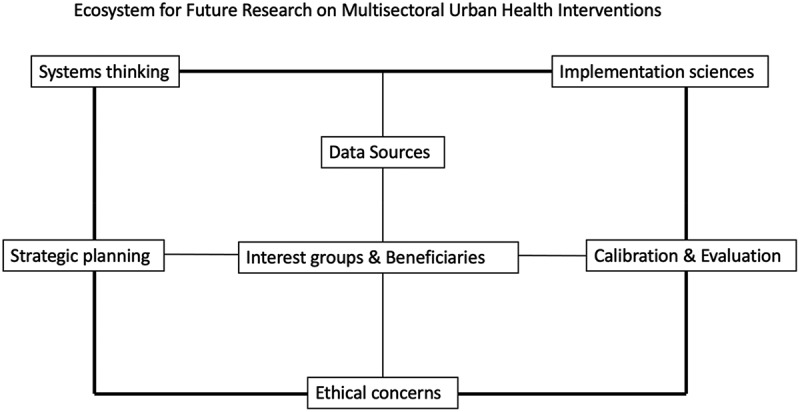
An ecosystem for future research on multisectoral health interventions.

Drawing from and integrating key identified principles, emergent research gaps, limitations and previously positioned recommendations, this section provides a framework that can guide future research into multisectoral interventions to improve urban health. This interconnected seven-component framework may further the current level of knowledge and development of urban health by addressing different components that are highly relevant (although not exclusive) to research on multi-sectoral interventions (see Figure 3).

Systems thinking (i.e. a holistic approach that focuses on how a system’s constituent parts interact and adapt): As urban populations grow across Africa and the urban health field evolves, the role of systems thinking will continue to change due to the different elements influencing urban health. For example, the use of sustainable urban street design interventions (i.e. where building location and natural ventilation improve air quality while encouraging more walking, like showcased in Burkina Faso [40]) will increase the ability to view cities as full-chain systems that affect health in positive and negative ways. Future research should focus on developing strategies for managing weak performing urban systems and for maintaining those that are human-centred, sustainable and cost efficient. Future research will also need to target factors influencing success and provide methods to effectively evaluate impact.

Implementation sciences (i.e. the use of strategies to adapt and apply evidence-based interventions to targeted settings): In this systematic review, there was a diversity of environments in which interventions were implemented, such as: churches, schools, local communities, workplaces, and health centres. Different methods pertaining to implementation sciences (e.g. effectiveness studies, research synthesis and mathematical modelling to embed evidence-based approaches to real-life programmes and policies) can further enquiry on multisectoral interventions. The external validity (i.e. to what extent can findings be generalised to other contexts or populations) of multisectoral interventions should be verified by wider stakeholder input and engagement processes as reported by some studies [47,56,73].

#### Data sources

The included studies illustrate that data on urban exposures and health-related records in cities can be generated and managed at multiple levels (e.g. patients, communities, service providers, organisations) but also by different sectors. The sectors identified in our review (see Table 5) act as building blocks to understand how multisectoral action towards improved urban health should be driven, and where possible, regulated following guidelines for health. Routinely and systematically collected data on target population and disease burden, pre-intervention, can be accessible through databases and can enable designing and implementation of comprehensive well-targeted multisectoral interventions such as the traffic collision database built in Kwazulu-Natal in 2013 [67]. The studies described the use of databases accessed by different groups, including interinstitutional authorities, local non-governmental organisations and civil society groups. With the integration of health technologies (e.g. smart devices, digital interventions or m-Health programmes) future research could focus on combining the information of multiple sources to get a comprehensive idea of the location and population where an intervention is targeted.

#### Strategic planning

Strategic planning was reported as a crucial component in the implementation of multisectoral interventions for urban health. Scholars should focus on factors that influence success and those that can hinder or facilitate efforts to co-design interventions with local communities. This systematic review shows that partners who can be a potential source of value for the deployment and evaluation of an intervention need to be identified early in the intervention design stages. This will prevent strategic issues related to resource constraints [55,58], administrative requirements [37,81] and uncertainty in effectiveness or uptake [41,63].

#### Interest groups & beneficiaries

One outcome of multisectoral interventions can be to provide more reliable disease burden data on interest groups and beneficiaries in cities. As shown in the selected articles, these groups span an array of individuals, from patients to researchers [60] and from students to professionals [46]. In the urban context, these individuals are likely to be affected by separate interventions across different sectors simultaneously. This may blur the boundaries between sectors that share similar objectives and targets, making role attribution and accountability harder in the context of multisectoral work. It is imperative that researchers identify beneficiaries that are most in need to ensure that interventions are targeted to those most in need and who will receive the most co-benefits.

#### Monitoring and evaluation

Monitoring and evaluation of interventions has direct benefits for implementation, particularly in terms of reaching vulnerable groups. Some studies have highlighted the importance of evaluation for health equity and justice, considering that the urban poor suffer most from the externalities of environmental degradation and fast urbanisation. A few studies have reported on the scarcity of good evaluation methods for calibrating multisectoral interventions and further scaling them to other settings or populations [55,73]. Without evaluation, it is challenging to identify and develop more cost-effective strategies for intervention implementation, particularly in fast urbanising contexts where strategic planning is often missing.

#### Ethical concerns

Data integrity (including data sharing on the effectiveness of interventions) should be openly and publicly available – particularly to its beneficiaries and the implementation of multisectoral intervention should be preserved in accordance with ethical guidelines. Researchers should focus on understanding the advantages and costs of multisectoral work. This will support in identifying and neutralising critical barriers that may affect the widespread effectiveness of interventions while protecting the beneficiaries. Scholars should consider multi- or trans-disciplinary methods to identify avenues for resolving ethical compliance, even more so in multi-national contexts. Ethical concerns should also extend to power dynamics between those designing the interventions, who bring the funding in, and the groups interventions are targeted at.

### Quality appraisal

The non-uniformity of the papers and disciplines contributed to heterogeneity in the extracted data. Six of the studies met the CASP appraisal criteria precisely and thus were considered the richest of the papers considered [30,60,74,75,77,81]. Across the studies aiming to assess the impact of multisectoral interventions, only a few contained sufficiently long follow-up periods. Another insufficiently considered factor was the possibility of confounders and bias in quantitative studies and relatedly, the consideration of the positionality of the researcher and its potential impact on qualitative research responses.

**Table 7.** Description of the included studies.

Author	Study design/methods	Country	City or region	State of initiative	Setting	Outcome targeted	Sectors involved
*Aaron et al. [30]	Cohort study	Ghana	East Mamprusi District, Nsawam, Suhum, Asamankese	Forming collaboration, monitoring or evaluation	Local community	Improving health behaviours, improving health outcomes, developing collaborations, providing social infrastructure to improve participation and empowerment of community members, improving access to services and physical infrastructure	Education, governance, health, marketing
Abdelazim et al. [31]	Cross-sectional	Egypt	Port Said City	Implementation	Health centre	Improving health behaviours (decrease smoking), health education (smoking cessation counselling)	Education, health
Adam et al. [32]	*Not mentioned*	South Africa	Western Cape	Planning project	Local community	Improving health behaviours (exclusive breastfeeding), improving health outcomes (reducing maternal, new-born and child mortality)	Academia or research, arts, community, non-profit, media
Adegun [33]	Mixed-methods; in-depth semi-structured interviews	South Africa	Kya Sands	Monitoring or evaluation	Local community	Improving the built and natural environment (improve knowledge on greening)	Community, education
Adongo et al. [34]	Qualitative participatory action research; interviews, field notes	Ghana	Ga East Municipal District	Implementation	Local community	Promoting health equity within urban neighbourhoods (improve reproductive health and child survival)	Education, governance, health
Ahmed et al. [35]	Qualitative participatory research	Kenya	11 villages in Mathare, 3 in Kibera, 4 in Mukuru	Planning project, forming collaboration, implementation, monitoring or evaluation	Local community	Improving health behaviours (increased awareness of food safety), developing collaborations, improving the built environment (limiting structural challenges for food vendors), providing social infrastructure to improve participation and empowerment of community members (increased cooperation with food vendors)	Agriculture, development, health, infrastructure
Aseyo et al. [36]	Mixed-methods; structured observations, cross-sectional surveys, in-depth interviews, FGD	Kenya	Kisumu	Forming collaboration, implementation, monitoring or evaluation	Local community	Improving health behaviours, improving health outcomes, providing social infrastructure to improve participation and empowerment of community members	Academia or research, governance, health, development (international)
Asgary et al. [37]	Quantitative; screening	Ghana	Ga East District	Planning project, forming collaboration, implementation, monitoring or evaluation	Health centre	Improving health outcomes (efficacy of screening for cervical cancer), improving access to services and physical infrastructure (smartphone-based training for visual inspection of cervix)	Academia or research, health
Bonnet et al. [38]	Quantitative case study	Burkina Faso	Ouagadougou	Forming collaboration, implementation, monitoring or evaluation	Local community	Developing collaborations, improving the built environment (road traffic injuries surveillance)	Education, governance, health, law enforcement
Bradley & Phone [39]	Qualitative participatory research	South Africa	Khayelitsha	Planning project, forming collaboration, implementation,	Local community	Improving health behaviours (preventing hypertension and diabetes), improving health outcomes (promoting healthy lifestyles), providing social infrastructure to improve participation and empowerment of community members	Arts, community, health
Bruyeron et al. [40]	Mixed-methods; cross-sectional surveys, interviews	Madagascar, Burkina Faso	Fada N’Gourma	Planning project, forming collaboration, implementation, monitoring or evaluation	Local community	Improving health behaviour (improving nutritional status)	Community, development, governance, nutrition
Cilliers et al. [41]	Mixed-methods; interviews	South Africa	Ngaka Modiri Molema, Doctor Rose Segomotsi Mompati, Kenneth Kaunda and Bojanala	Monitoring or evaluation	Health centre	Improving the built and natural environment (increasing urban green infrastructure in the form of health clinic gardens)	Academia or research, agriculture, community, development, environment, health
Cockburn et al. [22]	Qualitative case study	South Africa	eThekwini	Forming a collaboration	Workplace	Providing social infrastructure to improve participation and empowerment of community members	Conservation, education, environment, governance
Draper et al. [42]	Quasi-experimental study; FGD, cross-sectional surveys	South Africa	Alexandra township	Implementation	School or university	Improving health behaviours (improving physical fitness benchmarks in school-aged children)	Academia or research, education, health, private
Draper et al. [43]	Mixed-methods; participatory action research, cross-sectional surveys, FGD	South Africa	*Not mentioned*	Implementation	Church	Improving health outcomes (reduction BP, BMI, WC), improving health behaviour (physical activity, diet), providing social infrastructure to improve participation and empowerment of community	Academia or research, governance, health, non-profit, religious
Drechsel *et al*. [44]	Mixed-methods; pre- and post- cross-sectional assessment, narrative description	Ghana, Sierra Leone, Nigeria	Accra, Ibadan, Freetown	Implementation, monitoring or evaluation	Local community	Improving the natural environment, developing collaborations (improving understanding of informal irrigation)	Agriculture, development
El-Sherif [45]	Qualitative; FGD, narrative descriptions	Egypt	Etay El-Baroud	Planning project, forming collaboration	Local community	Improving built environment, improving access to services and physical infrastructure (e.g. improving connectivity in the city)	Academia or research, governance, housing, transport
Elmes et al. [46]	Mixed-methods; participatory research (mapping/modelling), field notes, narrative descriptions	South Africa	Monwabisi Park	Forming collaboration	Local community	Developing collaborations, providing social infrastructure to improve participation and empowerment of community members (improved water and sanitation)	Governance, private, social services
Elshimy & Ragheb [47]	Qualitative case study; narrative descriptions	Egypt	Cairo	Monitoring or evaluation	Local community	Improving health outcomes, improving the built environment and natural environment, providing social infrastructure to improve participation and empowerment of community members, improving access to services and physical infrastructure (e.g. improved air quality, encouraging of active transport, prevent pollution)	Construction, development (international), education, governance, hospitality, retail, transport
Everett-Murphy et al. [48]	Mixed-methods; cross-sectional surveys, FGDs	South Africa	6 cities (not mentioned by name)	Planning project	Local community (disseminated books to households)	Improving health behaviours (healthy eating in accordance with government nutritional guidelines)	Academia or research, governance, health,
Fosso [49]	Report on project implementation	Namibia	Windhoek, Rundu	Implementation, monitoring or evaluation	Youth centre, agriculture centre	Providing social infrastructure to improve participation and empowerment of community members (improved physical activity and reduced diabetes), improving access to services and physical infrastructure	Agriculture, governance, nutrition
Futterman et al. [50]	Quantitative; pre- and post-cross-sectional surveys	South Africa	Gugulethu, Vanguard	Monitoring or evaluation	Health centre	Improving health behaviour (improving adherence to guidance for the prevention of PMTCT)	Academia or research, governance, health, NGO
Gallaher et al. [51]	Mixed-methods; cross-sectional survey, interviews	Kenya	Kibera	Monitoring or evaluation	Local community	Improving health behaviours, providing social infrastructure to improve participation and empowerment of community members (influence of sack gardening on food security)	Academia or research, agriculture, community
Gallaher et al. [52]	Qualitative; FGD	Kenya	Kibera	Monitoring or evaluation	Local community	Improving health outcomes, improving the natural environment, providing social infrastructure to improve participation and empowerment of community members, improving access to services and physical infrastructure (e.g. greening neighbourhood, improving air quality, enhancing biodiversity)	Agriculture, education
Gartner et al. 2006	Retrospective cohort study	Senegal	Keur Cheikh Ibra	Monitoring or evaluation	Foodbank or nutrition centre	Improving health outcomes (reducing deterioration of nutritional status of malnourished children), improving health behaviours (promote nutritionally beneficial behaviours in mothers)	Academia or research, community, finance, governance, health
Gartner et al. 2006	Multi-point cross-sectional	Senegal	Diourbel	Monitoring or evaluation	Foodbank or nutrition centre	Improving health outcomes (nutritional recovery), improving health behaviour (change in attitude of feeding practises)	Academia or research, governance (multilateral), nutrition
Gartner et al. [53]	Quasi-experimental pre- and post- cross sectional survey	Senegal	Keur Cheikh Ibra	Monitoring or evaluation	Foodbank or nutrition centre	Improving health outcomes (improve or stabilise nutritional status in children), improving health behaviours	Academia or research, agriculture, community, finance, health, governance
Gore [54]	Qualitative; field notes, semi structured interviews	Uganda, Kenya	Kampala, Nairobi	Forming a collaboration	Local community	Developing collaborations, providing social infrastructure to improve participation and empowerment of community members	Agriculture, governance
Hailemariam et al. [55]	Qualitative: key informant interviews, in-depth interviews, document review, observation checklist	Ethiopia	Addis Ababa	Implementation	Health centre	Improving health outcomes (improved urban health by reforming primary health care), improved access to health infrastructure	Academia or research, governance, health
Harpham & Few [56]	Qualitative; in-depth interviews, key informant interviews, document analysis	Tanzania	Dar es Salaam	Monitoring or evaluation	Health centre	Improving health outcomes, providing social infrastructure to improve participation and empowerment of community members, improving access to services and physical infrastructure	Education, governance, health
Mahomed et al. [57]	Cross-sectional study	South Africa	Dr Kenneth Kaunda District, West Rand Health District, Bushbuckridge subdistrict	Monitoring or evaluation	Health centre	Improving health behaviours, improving health outcomes, developing collaborations, improving access to services and physical infrastructure	Education, development (international), governance, health
Makadzange et al. [58]	Qualitative descriptive case study	Eswatini	Matsapha	Planning project, forming collaboration, implementation	Local community	Improving urban health equity including improving health outcomes, physical infrastructure and environment, social and human development (e.g. improving access to WASH, reducing infant mortality, maternal mortality)	Academia or research, governance, international organisation, NGO
Mbachu et al. [59]	Cross-sectional	Nigeria	Enugu, Nsukka	Monitoring or evaluation	Church	Improving health outcomes (preventing cervical cancer)	Academia or research, religious
*Meintjes et al. [60]	Qualitative; using logs from counselling sessions	South Africa	Hanover Park, Cape Town	Monitoring or evaluation	Health centre	Improving health outcomes (improved mental health for depressed and/or anxious women living in poverty)	Academia or research, health
Michael et al. [61]	Cross-sectional	Tanzania	Nyamagana, Magu	Implementation	Drug retail outlet	Improving health outcomes (improving control and treatment of hypertension)	Academia or research, health retail, non-profit
Moosa et al. [62]	Qualitative; ethnography, observational, FGD	South Africa	Johannesburg	Monitoring or evaluation	Health centre	Improving health outcomes (improving prevention and health promotion)	Academia or research, *not further specified*
Mujere [63]	*Not applicable*	Zimbabwe	Harare	Monitoring or evaluation	Local community	Improving health outcomes (household food security), improving access to services and physical infrastructure (uptake of urban agriculture)	Academia or research, agriculture, city planning, governance
Muyeba [64]	Cross-sectional household surveys	South Africa	Khayelitsha	Monitoring or evaluation	Local community	Improving the built environment, improving access to services and physical infrastructure (improving home ownership to improve health)	Governance, construction, education
Nadesan-Reddy & Knight [67]	Retrospective secondary analysis of interrupted time series	South Africa	Chatsworth, KwaMashu	Implementation	Local community	Improving the built environment (reducing road traffic accidents)	Education, governance, transport,
Oldewage-Theron et al. [68]	Mixed-methods; cross-sectional study, in-depth interviews, key informant interviews, field notes	South Africa	Eastern Free State, Vaal Region of Gauteng	Planning project, forming collaboration, implementation, monitoring or evaluation	Local community	Improving health behaviours (increasing vegetable and soy consumption), developing collaborations, providing social infrastructure to improve participation and empowerment of community members	Academia or research, community, governance, workplace
Peterson et al. [69]	Mixed-methods; cross-sectional surveys, key informant interviews	Nigeria	Lagos	Monitoring or evaluation	Bank	Improving health behaviour (improving health seeking behaviour), improving access to services (increase health insurance), developing collaborations	Academia or research, governance, development, finance, non-profit, private
Pridmore et al. [78]	Mixed-methods; participatory research, interviews, cross-sectional surveys,	Kenya	Chaani, Kongowea, Playa Ancha y Cordillera, Rodelillo y Placeres	Planning project, implementation, monitoring or evaluation	Local community, (pre) school or university	Improving health outcomes (nutritional status among children)	Agriculture, education, governance, health, social services
Puoane et al. [65]	Quantitative; pre- and post- written tests, field observations	South Africa	Cape Town	Implementation	School or university, local community	Improving health behaviours, developing collaborations, improving access to services and physical infrastructure (improved CVD screening)	Education, governance, health
*Roche et al. [77]	Qualitative; key informant interviews, FGD	Ethiopia	Amhara, Oromia	Monitoring or evaluation	Foodbank or nutrition centre	*Not mentioned*	*Not mentioned*
*Sachikonye et al. [81]	Qualitative; in-depth interviews, semi-structured interviews	South Africa	Zandspruit, Johannesburg	Monitoring or evaluation	Local community	Improving the natural environment (trees preserving and protecting urban forest to mitigate climate variability), improving health behaviours (providing fruit trees as source of food and income)	*Not mentioned*
Schouw et al. [66]	Qualitative participatory research	South Africa	Western Cape	Planning project	Workplace	Improving work environment	Education, energy, health
Slingers & De Villiers [70]	Cross-sectional pre- and post- cross-sectional survey	South Africa	Cape Town	Monitoring or evaluation	Health centre	Improving health outcomes (improving hypertension management)	Education, health
Stern & Green [71]	Qualitative; in-depth interviews, observations	South Africa, United Kingdom	*Not mentioned*	Implementation	Local community	Improving urban health	Community, development, education, health, social services
Sun et al. [72]	Qualitative; in-depth interviews	Kenya	Nyeri	Implementation (pilot)	Local community, restaurant	Improving food environment (increasing access to healthy street foods)	Community, development, education, private
Tafesse et al. [73]	Cross-sectional study	Ethiopia	Addis Ababa	Monitoring or evaluation	Local community	Improve health outcomes, improving health behaviours	Education, governance, health
*van de Vijver et al. [74]	Qualitative; narrative case study	Kenya	Korogocho, Viwandani	Planning project, implementation	Local community, health centre	Improving health outcomes, providing social infrastructure to improve participation and empowerment of community members (cost-effective CVD prevention programme)	Communication, health, governance
*Warshawsky [75]	Qualitative; in-depth interviews	South Africa	Jozini	Monitoring or evaluation	Foodbank or nutrition centre Local community	Providing social infrastructure to improve participation and empowerment of community members, improve health behaviours (reduce food insecurity)	*Not mentioned*

Abbreviations: **BMI**, body mass index; **BP**, blood pressure; **CVD**; cardiovascular disease; **FGD**, focus group discussions; **NGO**, non-governmental organisation; **PMTCT**, prevention of mother to child transmission; **WASH**, water, sanitation and health; **WC**, waist circumference. * studies meeting CASP appraisal precisely.

## Discussion

The study performed a systematic review of the literature on multisectoral interventions in urban Africa to address NCD risks. It presents evidence and prospective potential of multisectoral interventions to increase urban health in Africa. For this purpose, five broadly framed research questions were posed. Our findings indicate the need for a broader range of studies in different African cities, considering that only 27.8% of African countries in the African Union were included, with over half of these studies being conducted in South Africa and Kenya. Most interventions were targeted at major cities within specific countries (Cape Town, South Africa and Nairobi, Kenya). In total, 22 sectors underpinned 52 studies, five sectors were present at all intervention stages: academia or research, agriculture, government, health, and non-governmental. We found nine guiding principles (Table 5), six health outcomes of interest (Table 6), and no measurement of the impact of the interventions on these outcomes. A summary of the primary research themes allowed us to identify crucial factors such as administrative factors, norms and power dynamics, and resource allocation that can influence the success of multisectoral interventions. The final research question focused on the potential areas where future research in urban health could offer significant insight. This question was addressed by integrating insights from the previous questions into a single, synthesised framework with seven components that may critically guide further development of the urban health field (Figure 3).

### Contribution to current knowledge

This study highlights an important evidence gap in the evaluation of impact on health outcomes (*n* = 0), hence urges for long-term methods for impact evaluation of multisectoral interventions [84]. Oni et al. (2020) have noted that the evaluation of interventions in rapidly urbanising cities in Africa requires looking beyond their immediate outcomes and instead considering their long-term impacts [85]. This can account for both positive and negative externalities that may arise. This is also reiterated by Gargani & McLean who draw attention to the complexity of intervention implementation in real-life contexts, particularly at scale. They recommend the principle of dynamic evaluations [84], which uses continuous and adaptive evaluation metrics to accommodate the way the impacts of interventions change over time and with scale.

This study illustrates how multisectoral partnerships with strong community engagement components and that work with existing capacity in local communities and the health system can support intervention delivery, as well as support securing resources, and political will for long-term sustenance of interventions. Recently, in the context of the COVID-19 pandemic in African cities, other research has noted that partnerships between actors, community volunteers, the private sector, and grassroots volunteers were critical for improving urban residents’ access to food [86]. Dynamic evaluations of such partnerships could help inform proper ways to design multisectoral partnerships that can address NCDs in African cities effectively in the long term. This study further highlighted the need for stronger documentation of multisectoral interventions that address potential biases, confounding and use research approaches that adequately consider the positionality between researchers and target populations. Foley et al. (2020) also likewise drew attention to the need for stronger rigour in how interventions in African cities are documented [87]. The issue around who runs and who publishes this documentation is also useful to highlight. In this study, we show that nearly half of the first authors are located outside the African continent (Table 3), raising important concerns around the disparities and inequities that may hinder research ownership, technical capacity of teams and evaluation of interventions by local groups. Approaches to strengthening both the quantitative and the qualitative approaches, and local technical capacity for designing, evaluating and reporting on multisectoral interventions on the continent are vital.

### Implications for policy and practice

Given that Africa continues to rapidly urbanise, multisectoral interventions gather various partners to address multiple systems impacting health outcomes in the context of complex urban challenges [7]. In the context of NCDs, this includes systems that improve access to living in clean environments, eat healthily, prevent injuries, engage in safe and inclusive physical activity, and achieve and maintain optimal mental health. Being cross-cutting across systems, such multisectoral interventions address the ‘*causes of the causes*’ of disease.

In addition to immediate impacts on health outcomes, there is increasing recognition of the centrality and catalytic potential for public health to integrate with various development agendas for the achievement of planetary health [88]. Global goals and targets around human rights, infrastructure development, sustainable development, gender equality, participation of people with disabilities and climate action in line with climate mitigation and adaptation strategies to gain positive externalities from multisectoral interventions targeting urban health and wellbeing. The health sector, which (alongside governments and technical health experts) has already been identified as a driver of multisectoral initiatives, should be supported with the resources to intersect their goals for NCD prevention with the shared goals of several global and national development agendas including the SDG 2030 Agenda. This may help to generate more resources to provide the administrative, financial, and training support which is critical for the success of multisectoral initiatives. Co-creation approaches and mindsets may fuel the process for other sectors to receive and reserve support and resources to participate in multisectoral partnerships.

Through our stakeholder engagement workshops, we found that urban practitioners desire practice-focused research and opportunities to share knowledge, failures, and successes and to interface with multiple sectors to achieve shared goals. This study is one such effort to fill that gap. Through future similar research and forums, best practices around community engagement, financing, addressing of perceptions, implementation, design, advocacy, and evaluation can be shared. Documentation can be supported through setting up learning networks, the exchange of knowledge, and the use of dynamic evaluation approaches to support more rigorous evaluations of interventions. Stakeholder engagement is an effective way of ensuring the integration of partnerships for the long-term success of multisectoral initiatives [39,55,57]. More partnerships with governments and exchanges between governments at the city and national level can help to shed light on factors that drive the integration of promising multi-sectoral initiatives and how governments and communities can be supported in this process.

### Implications for future research

Based on the findings of this systematic review, we recommend that future research generates more evidence on multisectoral initiatives in African cities with an emphasis on increased representation of countries, cities, settings, and health outcomes across the continent. Our results show that only 2% of all studies (Table 6) focus on health equity and that 47% of all articles are published by first authors (Table 3) who are not located on the continent. This underlines the need for research to be both focused on improving health equitably and to be conducted equitably. Such endeavour can take the form of formative research to improve existing high-impact interventions, as well as better support for the documentation of existing multisectoral initiatives. The ways in which these interventions are documented are important in gaining a better sense of how sectors collaboratively work towards improved urban health. One way forward would be to focus on the varying degrees of partnership that enable sectors to consult, collaborate and potentially integrate, their approaches and outputs.

Stronger measurement and evaluation of multisectoral interventions is needed. This should involve formative research that explores the factors behind the design of successful interventions and the design of metrics to support learning from successful multisectoral interventions. It should also capture components, partners, process-based factors, such as administrative considerations, incentives and efforts, to engage local communities. It is crucial to understand how multisectoral interventions work beyond their initial implementation or short-term feasibility testing. Long-term evaluative efforts should explore factors that enhance or militate against intervention sustainability. Particular attention should be paid to vulnerable and mobile urban populations who are hardly reached in follow-up. Other factors to be explored include different financing components of multisectoral interventions that can support their long-term delivery. One example would be to look at the kinds of incentives that can support equity in collaborations with private sector service providers and the resource arrangements that will strengthen instead of draining the capacity of community health workers and volunteers. This can also point towards research exploring the power dynamics between various multisectoral partners (e.g. between government and community representatives or global and local non-profit players) which subsequently can help to inform more beneficial equitable designs of multisectoral initiatives.

### Strengths and limitations

To our knowledge, this is the first systematic review to consider multisectoral interventions for the prevention of NCDs in urban Africa. We were able to consider the literature by exploring different settings, components, funders, players, and impacts of such multisectoral initiatives, which allowed the translation of findings into practical recommendations for research and action, as well as transdisciplinary research linking both endeavours. While heterogeneous forms of data can limit the use of more traditionally precise synthesis approaches based on more homogenised data (e.g. meta-analyses), we also consider this as strength of our study as it allows us to integrate evidence from different types of interventions. Our operational team was diverse, involving academics and practitioners who originated from, lived in and/or were working on urban health issues in a wide range of African countries supporting the contextualisation and proper interpretation of the research findings. Furthermore, our engagements with diverse stakeholders such as academics, civil society partners, government and other decision makers working on urban health issues in African cities also helped us refine our research questions and study the design and interpretation of subsequent findings. We recognise that this review provides the tip of the iceberg of the interventions that occur in formal urban areas and looks primarily at peer-reviewed outputs and works that academics are interested in, while academics are just one of many sectors involved in multisectoral interventions. We hope that our work will encourage more efforts to learn from and document a wide diversity of multisectoral interventions for urban health such as these to foster learning. Our recommendations for designing these interventions can support improved design, evaluation, and their documentation.

## Conclusion

Multisectoral initiatives can help to equitably improve the health of the public in rapidly growing African cities. There is a need for a wider range of multisectoral initiatives within African cities and across African countries. Existing evidence on such interventions shows that multisectoral initiatives guided by the principles of community health equity are dominant, with academic, government, community and non-governmental organisation partners playing a significant role in their design and evaluation. Beyond evaluating their initial feasibility, a stronger focus needs to be placed on the long-term success of multisectoral initiatives. Improving regional representation and considerations for bias and long-term impact will improve the design and impact of multisectoral interventions and therefore strengthen the inferences that can be made from them. This will require longer-term evaluations, as well as a stronger consideration of the power dynamics, resources available, and community preferences.
